# Acknowledgement to Reviewers of *Materials* in 2014

**DOI:** 10.3390/ma8010193

**Published:** 2015-01-07

**Authors:** Materials Editorial Office

**Affiliations:** MDPI AG, Klybeckstrasse 64, CH-4057 Basel, Switzerland

The editors of *Materials* would like to express their sincere gratitude to the following reviewers for assessing manuscripts in 2014:

Abate, Antonio
Abdallh, A. M. A.
Abdelmalak, Michael Naguib
Abe, Kentaro
Abenojar, J.
Abraham, Wolf-Rainer
Abu-Lebdeh, Yaser
Acet, Mehemet
Aggelis, Dimitrios G.
Agostini, Elisa
Agüero, Alina
Ahn, Yonghyun
Ajersch, Frank
Åkermo, Malin
Aktas, Z.
Al-Abadleh, Hind
Alam, M. Shahria
Albadarin, Ahmad B.
Albin, Sacharia
Alexis, Frank
Allan, Peter
Allinson, David
Almeraya, Facundo
Alonso-Lomillo, M. Asunción
Al-Tabbaa, Abir
Amand, Thierry
Amineh, Reza Khalaj
Ando, Takashi
Andrade, Carmen
Anfodillo, Tommaso
Angelescu, Daniel
Antic, Aleks
António, J.
Anzai, Jun-ichi
Aoki, Hiroyuki
Apostolopoulos, Georgios
Arenillas, Ana
Argyros, Alexander
Arima, Kenta
Arlazarov, Artem
Armentano, I.
Arnold, Donna C.
Aronne, Antonio
Ashraf, Shahid
Ashrafi, Behnam
Asquith, David
Ata, Athar
Avolio, Alberto P.
Azenha, Miguel
Azmi, A. I.
Bai, Jinbo
Baker, Robert
Balázsi, Csaba
Ban, Takayuki
Banerjee, Raj
Bao, Ying
Baragetti, Sergio
Barandiaran, Jose Manuel
Barberá, Joaquín
Barbuta, Costel
Barford, John Patrick
Barhoum, Ahmed
Barkoula, N.-M.
Bartlett, Rebecca
Baskoutas, Sotirios
Bastidas, J. M.
Bastos, Alexandre
Bastos, Ivan Napoleão
Bates, David
Baumeister, Joachim
Baweja, Daksh
Baxavanis, Theocharis
Bednarkiewicz, Artur
Beller, Daniel
Bellucci, Francesco
Belmonte, Thierry
Belviso, Claudia
Ben Khalifa, Nooman
Benali, Samira
Bencardino, Francesco
Berardi, Valentino Paolo
Berger, Walter
Berglund, Lars
Bermingham, M. J.
Bernardo, Enrico
Bhargava, Suresh Kumar
Bhattacharya, Rahul
Bian, Liming
Bikiaris, Dimitrios
Bikowski, André
Bilotti, Emiliano
Bing, Kong Ling
Birosca, Soran
Boesel, Luciano
Bohner, Marc
Boissiere, Cedric
Bolotov, Leonid
Boningari, Thirupathi Reddy
Boparai, Hardiljeet
Borges, João
Bouwmans, Géraud
Bozhilov, Krassimir N.
Bracke, Lieven
Branco, Fernando
Brandstätter, Christian
Brantley, William A.
Braun, Ulrike
Brennan, Anthony B.
Brischke, Christian
Brockman, Robert A.
Brüning, Karsten
Burkel, Eberhard
Burnley, Stephen
Byun, Joon-Hyung
Cabeza, Aurelio
Cachim, Paulo B.
Calzada, M. Lourdes
Cambier, Francis
Camblor, Miguel A.
Cammidge, Andrew
Campanelli, Sabina Luisa
Campbell, John
Çamurlu, H. Erdem
Cao, G.
Capobianco, John A.
Caporali, Stefano
Carbone, Giuseppe
Cardea, S.
Casalino, Giuseppe
Castanho, Miguel
Castela, A. S.
Castro-Gomes, Joao
Catellani, Marinella
Cavaliere, P.
Cavalli, Andrea
Cavalline, Tara
Cavallini, Mauro
Celano, Umberto
Cesano, Federico
Chadov, Stanislav
Chagnon, Gregory
Chamos, A. N.
Chan, Kwong Yu
Chang, Chih-hung
Chang, Chih-yuan
Chang, Jeong Ho
Chang, S. Y.
Chang-Liao, Kuei-shu
Changyun, Jiang
Chapman, James
Chatel, Gregory
Chatterjee, Sabornie
Chaudhry, Abdul J.
Chen, Changle
Chen, Chih-Ping
Chen, Chun-Hu
Chen, Jem-Kun
Chen, Jian
Chen, Kew-Yu
Chen, Ruei-Tang
Chen, T. M.
Chen, Xiaolong
Chen, Yangyin
Chen, Yue
Chen, Zeng-tao
Chen, Zuliang
Chenal, Jean-Marc
Chiechi, Ryan C.
Chien, Sum Tze
Chigrinov, Vladimir G.
Chipara, Dorina
Chiu, Kong Hwa
Chmielus, Markus
Cho, Man-Gi
Cho, Won-Ju
Choi, Duck-Kyun
Choi, Jae-Hong
Choi, Yoon-Seok
Chowdhury, Sanjib
Christodoulou, C.
Chu, Dewei
Chu, Jian
Chung, Chanmoon
Chung, T. C. M.
Ciardelli, Francesco
Ciardelli, Gianluca
Cinelli, Patrizia
Clarke, Tracey M.
Coakley, Eoin
Cobo, Alfonso
Cochran, Eric W.
Cole, Matt
Coll, Mariona
Collins, Frank G.
Cook, Wayne D.
Copeland, Audrey
Corcione, Carola Esposito
Cordoyiannis, George
Corma, Avelino
Coronas, Joaquín
Corriou, Jean-Pierre
Costa García, Agustín
Courtin-Nomade, Alexandra
Cousins, Brian G.
Cown, Dave J.
Cran, Marlene J.
Creatore, M.
Criado, M.
Cristiani, Pierangela
Cullen, Jonathan M.
Czerwinski, Frank
Dai, Jian Guo
Dalgarno, Kenneth
Damborenea, Juan
Daniel, Laurent
Dapino, Marcelo J.
Darling, Seth
Das, Diganta B.
Das, Suman
Datsyuk, Vitaliy
Daus, Birgit
Davidovits, Joseph
Davidson, Jane H.
Davies, Peter
Davim, Paulo
De Belie, Nele
De Brito, Jorge
De Geest, Bart
De Rooij, Mario
De Sio, Luciano
De Tommasi, Domenico
Defouw, John
Delale, Feridun
Deleporte, Emmanuelle
Delgado, Rafael
Deng, Hongbing
Deng, Shiqiang
Devaraj, Amutha
Dewan, M. Washim
Dhere, Neelkanth G.
Diaz, Urbano
Dickinson, Alex
Didangelos, Athanasios
Diéguez, Oswaldo
Dietrich, D.
Dimiev, Ayrat M.
Dimitrijevic, Nada M.
Dinia, Aziz
Distaso, Monica
Djurdjevic, Mile B.
Dobrzański, L. A.
Domenek, Sandra
Domenici, Valentina
Dong, F.
Dong, Hongbiao
Dong, Liang
Dorozhkin, Sergey V.
Douki, Thierry
Dourado, Fernando O. Queirós
Dragic, Peter
Dragoni, Eugenio
Driver, Julian
Duarte, Ana Rita C.
Dubey, Ashish
Dufossé, Laurent
Dujardin, Christophe
Dunn, J. B.
Duong, Hai M.
Dupin, Jean Charles
Eamon, Christopher
Easton, Mark
Ebara, Ryuichiro
Ebel, Thomas
Ebendorff-Heidepriem, Heike
Ebong, Abasifreke
Edalati, Kaveh
Efthymiou, Evangelos
Eick, Sigrun
Eizenberg, Moshe
Eklund, Per
Elaissari, Abdelhamid
Elbaz, Lior
El-Kaderi, Hani M.
Elliman, Robert
Elliott, Simon
Elsentriecy, Hassan
Endres, Frank
Enfedaque, Alejandro
Engelberg, Dirk
Engler, Olaf
Eramo, Giacomo
Erathodiyil, Nandanan
Erdem, Emre
Espallargas, García S. J.
Espino, Daniel M.
Esposito, Emanuela
Evangelista, Luís
Evlyukhin, Andrey B.
Fabio Zuluaga, Hector
Fabregat, Azael
Fabrizio, Quadrini
Falletta, Ermelinda
Fedel, Mariangela
Feliu, Sebastian
Felser, Claudia
Feng, Xian
Fernandes, Emanuel M.
Fernandes, Susete
Fernandez, A. I.
Fernsler, Jonathan
Ferrari, Maurizio
Ferreira, José Maria da Fonte
Ferrier, E.
Ferro, Gabriel
Février, Sébastien
Fierro, J. L. G.
Filonovich, Sergej
Fletcher, Ashleigh
Focacci, Francesco
Fogelström, Linda
Follain, Nadège
Foot, Peter
Francesconi, Grazia M.
Fraternali, Fernando
Fratini, Livan
Fray, Derek J.
Friák, Martin
Fridkin, Vladimir
Frignani, Alessandro
Fujita, Kyoko
Fujita, Toyohisa
Fujiwara, Kohei
Fukada, Toshiyuki
Fukui, Kunihiro
Funahashi, Ryoji
Fürbeth, Wolfram
Furlani, Edward P.
Furue, Hirokazu
Gabbitas, Brian
Gaddam, Raghuveer
Galán-Mascarós, José Ramón
Gallego, Sergi
Galstian, Tigran
Ganguly, Supriyo
Gao, Junqi
García, Hermenegildo
García, Juan
Garcia-Escorial, A.
García-Etxarri, Aitzol
García-Gutiérrez, Mari-Cruz
Garratt, Elias
Garrett, David J.
Garrett, Simon J.
Geddis, Demetris L.
Gerber-Lemaire, Sandrine
Gernay, Thomas
Gerôme, F.
Gerós, Hernâni
Gervasini, Antonella
Gherardi Hein, Paulo Ricardo
Gheribi, Aimen E.
Ghiotti, A.
Ghosh, Gargi
Giannakos, Konstantinos
Gil, Francisco Javier
Giovambattista, Nicolas
Girousi, Stella
Gkotsis, Petros
Glaeser, J. A.
Godinho, Maria
Goel, Saurav
Gomez-Romero, Pedro
González, María de las Nieves
González, S.
González-Fonteboa, Belén
Gonzalez-Marcos, M. Pilar
Goodall, Russell
Goodhew, Steve
Gordon, Wong
Grajcar, A.
Granberg, Hjalmar
Grandini, Carlos R.
Granozio, Fabio Miletto
Grasso, Salvatore
Graule, Thomas
Gray, Kimberly A.
Greco, Antonio
Greenly, Justin M.
Gregg, J. M.
Gregorczyk, Keith
Grimaldi, C.
Grimsdale, Andrew Clive
Grujicic, M.
Guan, Dongsheng
Gubner, Rolf
Gueunier-Farret, M.
Guldiken, Rasim
Guldner, Norbert W.
Guo, Youguang
Guo, Zhanhu
Gupta, Nikhil
Gupta, Rishi
György, Enikö
Ha, Kiryong
Habert, Guillaume
Habijan, Tim
Haick, Hossam
Haider, Waseem
Hallberg, Håkan
Halle, Thorsten
Hamilton, Jeremy
Hamzaoui, Rabah
Han, Daewoo
Han, Min-Cheol
Han, Yousoo
Han, Zhao Jun
Hanaor, Dorian
Hanawa, Takao
Hane, Kazuhiro
Hangai, Yoshihiko
Hanna, Junichi
Hara, Masayuki
Harding, D. R. K.
Hargreaves, Justin
Harkin, Damien
Hassan, Marwa
Hassel, Achim Walter
Hattori, Haroldo T.
Havenith, Remco
Hayakawa, Tohru
Hayashi, Yoshihiko
Hayes, Daniel J.
He, Bo
Healy, Noel
Heath, Andrew
Heilmann, Andreas
Hellier, Alan Keith
Henniges, Ute
Herting, Gunilla
Hezel, Dominik
Hickey, Stephen G.
Hidalgo, J. Ignacio
Highland, Matthew J.
Hilliou, Loic
Hinderberger, Dariush
Hinds, G.
Hintze, Wolfgang
Hirama, Hirotada
Hiziroglu, Salim
Ho, Johnny Chung Yin
Ho, Wen-Fu
Holmström, Stefan
Hong, Cheng-Shong
Hong, Yi
Hossain, Khandaker M. A.
Hosur, Mahesh V.
Howard, Ian A.
Howarter, John
Hsiao, Vincent
Hsu, Hsiu-Fu
Hu, Jinlian
Hu, Jonathan
Hu, Ning
Hu, Yuh-Chung
Huang, Baoshan
Huang, Huang-Chiao
Huang, Jie
Huang, Mao-Jung
Huang, Wei Min
Huang, Yuanding
Huang, Yu-Tzu
Hubbe, Martin A.
Hubert, Madlen
Hubner, Jens
Hudhomme, Piétrick
Hug, Eric
Hughes, Taylor L.
Humbeeck, Jan Van
Hun Ryu, Sung
Hüttner, Sven
Hyun, Hoon
Ibana, Don
Ibris, Neluta
Iezzi, Giovanna
Ijima, Hiroyuki
Ingarao, Guiseppe
Ingham, Jason
Iniesta, Jesús
Inoue, Katsutoshi
Ionov, Leonid
Islam, Syed Kamrul
Ito, Seigo
Ivanov, Dimitri A.
Ivanov, Ilia N.
Iwasaki, Tomohiro
Jabbari, Mohammad Ali
Jabbarzadeh, Ahmad
Jackson, Nathan
Jakli, Antal
Jakubowicz, Ignacy
Jalali, Said
Janshoff, Andreas
Janus, Magdalena
Jayakumar, Rangasamy
Jayasinghe, Suwan
Jeng, Chyuan-hwan
Jeong, Doo Seok
Jeong, Han Mo
Jeong, Moon Seok
Jeong, Yongchae
Jestin, Jacques
Jezierska, Julia
Jiang, Jiang
Jiang, Liudi
Jin, Kejia
Jo, Young Min
Johnson, Scooter D.
Jones, Lathe
Jordon, Brian J.
Jose, Joby Kochumalayil
József, Karger-Kocsis
Jung, Sungmoon
Kadota, Kazunori
Kaewunruen, S.
Kako, Tetsuya
Kalaskar, Deepak
Kamegawa, Takashi
Kan, Daisuke
Kaneco, Satoshi
Kang, Chulhun
Kang, Lifeng
Kang, Thomas
Kannan, Bobby
Karalekas, Dimitris
Karg, Matthias
Kartsonakis, I. A.
Kasper, Kurtis
Katmis, Ferhat
Katsamenis, Orestis L.
Katta, Lakshmi
Kaufman, Laura J.
Kaufman, Michael
Kawanago, Takamasa
Kawashima, Shiho
Ke, Changhong
Kelley, Michael J.
Kepczynski, Mariusz
Kergoat, Loïg
Kessler, Horst
Kevern, John
Khaliq, Wasim
Kharlampieva, Eugenia
Khatib, Jamal
Khun, Nay Win
Kidoaki, Satoru
Kiefer, Björn
Kilian, Kristopher A.
Kim, Chang-Soo
Kim, Dae Eun
Kim, Do Hyun
Kim, Dong-Pyo
Kim, Doseok
Kim, Eunkyoung
Kim, Gap-Yong
Kim, Hak Yong
Kim, Hee Chan
Kim, Heesun
Kim, Hyeong Joon
Kim, Jae-Hoon
Kim, Jin Kuk
Kim, Jung Ho
Kim, Kwang S.
Kim, Seung-Joo
Kim, Taehwan
Kim, Yoong Ahm
Kimura, A.
Kimura, Munehiro
Kimura, Yoshiharu
Kindt, James T.
King, Michael R.
Kishi, Yoichi
Kivisäkk, Ulf
Klemperer, Walter G.
Klenk, Andreas
Klepka, Andrzej
Klose, Christian
Knight, Jonathan
Kobelke, Jens
Köferstein, Roberto
Kofinas, Peter
Kojima, Chie
Kolasinski, Kurt W.
Kolppo, Kari
Komnitsas, Kostas
Konegger, Thomas
Kontturi, Eero
Koodali, Ranjit
Koos, Antal A.
Korai, Hideaki
Koricho, Ermias Gebrekidan
Korposh, Serhiy
Korshunov, M. M.
Korstanje, Ties J.
Koseki, Toshihiko
Koskinen, Jari
Kotadia, Hiren
Kotobuki, Masashi
Koukouzas, Nikolaos
Kourkoumelis, Nikolaos
Koutsoukos, Petros
Kowalska, Ewa
Kraehmer, Andrea
Kranzmann, A.
Kriezis, Emmanouil E.
Krishnan, Sadagopan
Krupski, Aleksander
Kuder, Katherine G.
Kuehn, Christian
Kühn, Fritz E.
Kukli, Kaupo
Kuktaite, Ramune
Kulichikhin, Valery
Kunioka, Masao
Kuo, Chao-Yin
Kuo, Dong-Hau
Kuo, Shiao-Wei
Kuo, W. T.
Kuo, Y. K.
Kwitkowski, Lech
Kwok, C. T.
Kwon, Il-Keun
Kymissis, Ioannis
Kyzas, George Z.
Labergere, Carl
Labrincha, J. A.
Lai, Chao
Lai, Chao-Sung
Lalevée, Jacques
Lambert, Paul
Lamberti, Gaetano
Lammens, Nicolas
Lampke, Thomas
Lan, Wen-How
Langhals, Nicholas B.
Lannutti, John J.
Lantada, Andres Diaz
Lanzoni, Luca
Larobina, Domenico
Laschat, Sabine
Lattuada, Marco
Lavrentovich, Oleg D.
Law, David
Lazzara, Giuseppe
Le Gac, Pierre-Yves
Lebaron, Richard
Leblanc, Roger
Leclerc, Corey
Leclerc, Nicolas
Lee, Bongmook
Lee, Cheng-Kang
Lee, Cheol Jin
Lee, Dongwon
Lee, Jae-chun
Lee, Jehyun
Lee, Juliana Tsz Yan
Lee, Mi-Jung
Lee, Sang Bok
Lee, Seung Hee
Lee, W.
Lee, Woojin
Lee, Ya-Ju
Lehmann, Jean
Lehmhus, Dirk
Leinenbach, Christian
Lekakh, Simon
Leon, Marta
Letizia, Rosa
Leung, Pui Tak
Levänen, Erkki
Lévêque, Patrick
Lewis, Gladius
Li, Bin
Li, Dingsheng
Li, Gang
Li, Guo Liang
Li, Guoqiang
Li, Kang
Li, Luhua
Li, Qiliang
Li, Quan
Li, Robert K. Y.
Li, Weiyang
Li, Xiang
Li, Yang
Lianos, Panagiotis
Lichtensteiger, Céline
Liew, S. L.
Lin, Chhiu-Tsu
Lin, Dong
Lin, Hong-Ping
Lin, I.
Lin, Jiang-Jen
Lin, Jing-Jenn
Lin, Jui-Teng
Lin, Song
Lin, Wei-Ting
Lin, Yuqing
Ling, Tung-Chai
Lionetto, Francesca
Lira-Cantu, Monica
Liu, Bo-Tau
Liu, Chuan-Hsi
Liu, Fwu-Hsing
Liu, Hongfei
Liu, Hongwei
Liu, Jingfei
Liu, Jinxuan
Liu, Rui
Liu, Yongliang
Lo Presti, Davide
Loke, Vincent
Lokuge, Weena
Lombillo, Ignacio
Lonjon, Antoine
López, Enrique Vera
Lord, Megan S.
Lorenzo, Donati
Lorenzo, E.
Lorite, I.
Lousteau, Joris
Lu, Cheng
LU, Li
Lu, Weigang
Luches, Armando
Ludwig, Ralf
Lugo, M.
Luo, Yanhua
Luyckx, Geert
Luzio, Alessandro
Lvov, Yuri
Ma, Dexin
Ma, John Z.
Maassen, Jesse
Machida, Masashi
Maclachlan, Mark J.
Maeda, Tetsuro
Mafi, Arash
Magliulo, Maria
Mahajan, Sujit
Mahesh, B. V.
Maheshwari, Vivek
Maierhofer, Christiane
Major, Matthew J.
Mallavia, R.
Malucelli, Giulio
Mamba, Bhekie B.
Manciu, Felicia
Manfredi, Diego
Mangialardi, Teresa
Mani, Gopinath
Mann, Amanda K. P.
Margarido, Fernanda
Margulis, Walter
Markos, Christos
Markou, Giorgos
Marsal, Lluis F.
Martinelli, Enzo
Martínez, Adriana
Martins, Rodrigo
Martí-Vargas, J. R.
Marton, Zsolt
Masai, Hirokazu
Masami, Ikeyama
Mascher, Peter
Materer, Nicholas F.
Mathaudhu, Suveen N.
Mazzolani, Federico
McCloskey, Bryan D.
McDonald , Armando G.
Mecartney, Martha
Mehl, Georg
Meier, Urs
Meneghetti, Giovanni
Menendez, J. L.
Meng, Yi
Mercolini, Laura
Mertens, Anne
Meulenberg, Robert W.
Mhaede, Mansour
Michailidis, Νikolaos
Mihashi, Hirozo
Milanese, Daniel
Minteer, Shelley D.
Miomandre, Fabien
Mistakidis, Euripides S.
Mittal, Vikas
Mizutani, Fumio
Młyniec, A.
Mobed-Miremadi, Maryam
Mock, Adam
Möhwald, Helmuth
Mokaya, Robert
Molchan, I. S.
Molina, Pablo
Møller, Uffe
Monge, M. A.
Monteiro, Regina C. C.
Montemor, Maria Fátima
Monticelli, Cecilia
Moreira, M. Teresa
Moreno Tost, Ramón
Moriana, Rosana
Morkoc, Hadis
Morris, Michael
Mortensen, Andreas
Motz, Günter
Mou, Chung-Yuan
Mougenot, J.
Müller, Bert
Muller, Robert N.
Muller-Buschbaum, Peter
Munoz-Pinto, Dany
Murcia-López, S.
Musso, Simone
Mutin, Hubert
Naher, Sumsun
Nair, Lakshmi S.
Nair, Sriramya D.
Naji, Majd Al
Najm, Husam
Nakagaito, Norio
Nakayama, Masaharu
Nam, N. D.
Nam, S.
Nara, Yoshitaka
Naraghi, M.
Natali, Maria Elia
Naya, Shin-ichi
Neimark, Alexander V.
Neri, Giovanni
Nespoli, Adelaide
Ngai, T.
Nganga, Sara
Nien, H. H.
Nohut, Serkan
Nomura, Kotohiro
Novakovic, Jelica
Nunes, Sandra
O’Connor, É.
Obalová, L.
Obidziński, Sławomir
Odijk, Theo
Ogihara, Hitoshi
Ogino, Hiraku
Ogle, Kevin
Oh, Byung Hwan
Ohara, Keishi
Ohno, Hajime
Ohshima, Tatsuya
Okawa, Seigo
Okimoto, Yoichi
Oliva, Leone
Olmi, Giorgio
Onishi, Hiroshi
Onwudili, J. A.
Ordóñez, Salvador
Oskouei, Reza
Osório, Wislei R.
Osten, H. Jörg
Ou, Jianzhen
Oudah, Fadi
Pace, Andrea
Pacheco-Torgal, Fernando
Pan, Tongyan
Pan, Xuan
Pan, Zhongqi
Panagiotaras, Dionisios
Panesar, Daman
Pantazopoulos, George
Papadopoulou, K.
Papavassiliou, Dimitrios V.
Park, Soo-Jin
Park, Yeong-Joon
Parker, Sandra
Parsons, Jason
Parviz, Babak Amir
Paschalis, Eleftherios I.
Pasek, Matthew
Pasupathy, Praveen
Patenaude, Mathew
Patolsky, Fernando
Patten, John
Patzke, Greta Ricarda
Pavlidis, Ioannis
Peacock, Anna
Pedersen, Henrik
Pedone, Alfonso
Pehlken, Alexandra
Peisert, Heiko
Pellicer, Eva
Pelton, Robert H.
Peng, Bo
Pereira de Oliveira, L. A.
Perez, M.
Pérez, Marco Antonio
Peroukidis, Stavros D.
Perrot, Arnaud
Pervak, Volodymyr
Peters, Kara
Peterson, Karl
Petrlova, Jitka
Petruzzelli, Domenico
Peukert, Wolfgang
Pey, Kin Leong
Peyvandi, Amirpasha
Philipose, U.
Phillion, Andre
Photinos, Demetri
Pickett, Matthew D.
Pietrzyk, Bozena
Pilat, Francesco
Pingarron, Jose Maria
Pirker, Stefan
Piro, Benoit
Pita, Marcos
Plank, Johann
Plechkova, Natalia
Pokhrel, Suman
Pomianowski, Michal
Poncelet, Denis
Poon, S. J.
Popat, Ketul C.
Popov, Anatoliy V.
Populoh, Sascha
Portehault, David
Potgieter, Herman
Power, Ian M.
Prade, Rolf
Pradhan, Sangram K.
Prashanth, Konda G.
Pretsch, Thorsten
Prosandeev, Sergey
Provis, John L.
Pucci, Andrea
Puche, Regino Sáez
Puertas, Francisca
Puga, H.
Pujo, Mariacinta C.
Pullammanappallil, Pratap C.
Puppi, Dario
Qiao, Kun
Qiu, Liang
Quiquempois, Yves
Radhika, Madhav A.
Ragab, Kh. A.
Rahbar, Nima
Rahier, Hubert
Ramaker, David E.
Ramanujam, Prabhu
Ramesh, Aramandla
Ramirez, Anibal
Ramirez, Carmen
Rangan, Vijaya
Ras, Robin H. A.
Rasekh, Manoochehr
Ratna, Banahalli R.
Ravelli, Davide
Rawls, Henry
Ray, Asim
Rebelo, Susana L. H.
Reclaru, Lucien
Ree, Moonhor
Refait, Ph.
Reijnders, Lucas
Reinhardt, Hans-Wolf
Reis, P. N. B.
Reiss, Guenter
Ren, Yuhang
Requena, Guillermo Carlos
Rhamdhani, M. Akbar
Ribeiro, Maria S.
Ribot, François
Riccardi, Leonardo
Richards, Bryce S.
Richardson, Hugh H.
Riggs, Brian C.
Ritala, Mikko
Ritcey, Anna
Rob Sharif, Muhammad Ali
Robertson, Peter K.
Robinson, Sara C.
Rodrigues, Danieli
Rodríguez, M. A.
Rodríguez-Valverde, M. A.
Roeb, Martin
Roggendorf, Hans
Rolon-Garrido, Victor
Romanato, Filippo
Rometsch, Paul
Ronca, S.
Ronkainen, Niina J.
Ros, Blanca M.
Rossi, Claire
Rost, Marcel J.
Rouabhia, Mahmoud
Rowland, Doug
Rowson, Neil
Roy, Anish
Ruhl, Aki Sebastian
Russell, Richard
Ruys, Andrew
Ryan, James G.
Sa´ncheza, Luis
Sabo, Ronald
Sadeghi, Alireza
Saleh, Haitham
Salvador, James
Sanchez, Florence
Sánchez-Ferrer, Antoni
Sánchez-Jiménez, Pedro E.
Sandri, Giuseppina
Santana, Juan J.
Santos, Catarina
Sardella, Eloisa
Sato, Soshi
Sawpan, Moyeenuddin A.
Sazio, Pier
Schaefer, Dirk J.
Schlangen, Erik
Schledjewski, R.
Schmidt, Daniel F.
Schneider, Raphael
Schneider, Raphaël
Schultz, B. F.
Schulze, Ulrich
Schuman, Thomas
Schwarzkopf, J.
Scotognella, Francesco
Scott, Jason
Sedev, Rossen
Segawa, Hiroyo
Seguí Palmer, Concepción
Seifalian, Alexander
Seifert, Gerhard
Seisenbaeva, Gulaim A.
Semba, Takeshi
Sen, Tushar
Seppälä, Jukka
Sepúlveda-Escribano, Antonio
Sesana, Raffaella
Shafaei, Shahram
Shah, Ramille
Sharif-Khodaei, Zahra
Shehata, Medhat
Shen, Li
Sherratt, Michael J.
Shi, Xianming
Shian, Samuel
Shibasaki-Kitakawa, Naomi
Shih, Po-Jen
Shimosawa, Tatsuo
Shin, Hyung Shik
Shinagawa, Tsutomu
Shing Ching, Tak
Shiraishi, Yasuhiro
Shiue, Sham-Tsong
Shokuhfar, Tolou
Shon, In-Jin
Shtein, Max
Shubhakar, Kalya
Sidiras, Dimitris
Siegel, Jakub
Sierra, Teresa
Silberschmidt, Vadim
Sillanpaa, Mika
Silva, H. M. Ribeiro Dias da
Silva, Rui A.
Silveyra, Josefina
Simar, Aude
Simka, Wojciech
Simões, Sónia
Singh, Mahi
Singh, Ranjan
Sinha, Arijit
Sista, Prakash
Skirtach, André G.
Slowing, Igor I
Sluckin, Tim J.
Slussarenko, Sergei
Smått, Jan-Henrik
Smay, Jim E.
Smith, Mark
Sobolkina, Anastasia
Södergård, Anders
Söderholm, Patrik
Soin, Navneet
Soldano, Caterina
Sommer, Michael
Song, Fei
Song, Yi
Sorgente, Donato
Soroushian, Parviz
Sorrentino, Andrea
Southam, Gordon
Spadea, Saverio
Spiesz, Przemek
Spigarelli, Stefano
Spychaj, Tadeusz
Srinivasa, Arun
Srivastava, Alok M.
Stannarius, Ralf
Steinbach, Ingo
Stepuk, Oleksandr
Stevenson, Adam J.
Stokes, Kevin L.
Stolichnov, Igor
Strangwood, Martin
Straumal, Boris
Straumal, Boris B.
Streli, Christina
Strömme, Maria
Strzhemechny, Yuri
Stuhr, Uwe
Stumpe, Joachim
Su, Lihong
Suárez, Isaac
Suarez-Garcia, Fabian
Sue, Hung-Jue
Sugden, Kate
Sukhishvili, Svetlana
Sumner, Joy
Sun, Henghu
Sun, Hongqi
Sun, Kien Wen
Sun, Shi-Peng
Sun, Tao
Sun, Xiao
Sunol, Joan Josep
Suryanarayana, C.
Suryanto, Benny
Sutor, Alexander
Suzuki, Daisuke
Swain, Michael
Swaminathan, Viswanathan
Światowska, Jolanta
Syrrokostas, G.
Sytschev, Alexander E.
Tabrizian, Roozbeh
Taglietti, Angelo
Tailhades, Philippe
Takamura, Yayoi
Takeuchi, Masato
Tamai, Toshiyuki
Tanaka, Keiji
Tang, Luping
Tang, Ning
Taratula, Oleh
Tariq, Mohammad
Tauer, Johannes
Taylor, Doris
Teixeira, Fernando
Tena-Zaera, Ramon
Teng, Hsisheng
Teoh, Lay Gaik
Terentjev, Eugene M.
Theivasanthi, T.
Thomas, C.
Thomas, Carlos
Thomas, Stuart
Thöming, Jorg
Thompson, Van P.
Thomson, Andrew
Tian, Yanqing
Tigli, Onur
Tisserat, Brent
Tiwari, Shruti
Tjong, S. C.
Tkacz-Smiech, Katarzyna
Todaro, Simona
Tofail, Syed A. M.
Tokoro, Chiharu
Tomashchuk, Iryna
Tomich, John
Tomlinson, Douglas
Torimoto, Tsukasa
Torrenti, Jean Michel
Toupance, Thierry
Traisnel, Michel
Tricot, Gregory
Trimmel, Gregor
Tripp, Carl P.
Troles, Johann
Tsao, Chung-Chen
Tsao, L. C.
Tsay, Chien-Yie
Tsay, L. W.
Tse, Ming-Leung Vincent
Tsivilis, S.
Tsouknidas, A.
Tsubota, Toshiki
Tsujimoto, Takashi
Tuch, Bernard E.
Tukker, Arnold
Tulliani, Jean-Marc
Tulunoglu, Ibrahim
Tung, Tran Thanh
Tung, Vincent C.
Uher, Ctirad
Uji-I, Hiroshi
Umeton, Cesare
Upadhyay, Piyush
Uraoka, Yukiharu
Uygun, Basak E.
Vaiano, Vincenzo
Vairo, Giuseppe
Valente, Isabel
Valentini, Luca
Van den Eeckhout, Koen
Van Gemert, Dionys
Van Riessen, Arie
Van Stam, Jan
Van Tittelboom, Kim
Van Velzen, Ulphard Thoden
Vannier, Rose-Noëlle
Vardaxoglou, Yiannis
Várnai, Anikó
Vázquez, Enric
Vedrine, Jacques
Venkataraman, Shrinivas
Verge, Pierre
Vicente, J. de
Vigier, Karine
Víllora, Gloria
Virtanen, Sannakaisa
Visai, Livia
Vizcaíno, Arturo J.
Vladimirov, Ivaylo
Vodenitcharova, Tania
Vogel, Michael
Vogtländer, Joost
Voisey, K. T.
Von der Mark, Klaus
Vonau, W.
Vorndran, Elke
Vos, Paul de
Vozmediano, María
Vygranenko, Y.
Wagner, Travis
Waite, Matthew M.
Wallinder, Inger Odnevall
Wan, Kai Tai
Wan, Kai-Tak
Wang, Ce
Wang, Chien-Lung
Wang, Chong-Min
Wang, Li
Wang, Min
Wang, Ran
Wang, Shanfeng
Wang, Shaobin
Wang, Xiao-Qian
Wang, Xungai
Wang, Zhonglin
Ward, L. P.
Ward, Liam
Watts, Benjamin
Wei, Suying
Weimer, Paul J.
Weinstein, Dana
Weiss, Jason
Wennerber, Ann
Whang, Wha-tzong
White, Timothy
Wille, Kay
Williams, Vance E.
Wilson, Mark. R.
Wiskott, Anselm H. W.
Witt-Enderby, Paula A.
Wondraczek, Karin
Wong, Danny K. Y.
Wu, C. H.
Wu, Jiamin
Wu, Kevin
Wu, Mao-Sung
Wu, Shin-Tson
Wu, X. F.
Wu, Xijia
Wurmehl, S.
Xia, Wei
Xue, Wei
Yamabe-Mitarai, Yoko
Yamamoto, Akio
Yamamoto, Go
Yamauchi, Satoshi
Yamauchi, Yusuke
Yan, Ning
Yan, Xiaoli
Yang, H. Y.
Yang, Jinglei
Yang, Kap Seung
Yang, Renbo
Yang, Yong
Yannick, Mugnier
Yasuzawa, Mikito
Yazyev, Oleg
Yeh, Jui-Ming
Yeh, Min-long
Yentekakis, I. V.
Yeow, Edwin K. L.
Yi, Jiabao
Ying, Shang-Ping
Yokoi, Toshiyuki
Yokoyama, Kenji
Yoo, Dong Jin
Yoon, Seong-Ho
Yoon, Ung Chan
Yoshinari, Masao
Yoshino, Toshihiko
Yoshitake, Hideaki
Yousif, Belal F.
Yu, Chang-Feng
Yu, Dingshan
Yu, Long
Yu, Yanghai
Yun, Sang-Won
Zakutayev, Andriy
Zaleski, Jeffrey
Zanetti, Mariachiara
Zaraska, Leszek
Zehetner, Christian
Zeinert, Andreas
Zeugolis, Dimitrios
Zhang, Lifeng
Zhang, Mingxing
Zhang, Xu
Zhang, Yan
Zhang, Zuhua
Zheng, Wei
Zhou, Hongyu
Zhou, Jinming
Zhou, Jun
Zhu, Weihong
Zhu, Xiaoshan
Zhu, Youfeng
Zhu, Yu
Zhuravleva, Ksenia
Zimmermann, Uwe
Zitoune, Redouane
Zomorodian, A.
Zou, Ying
Zubarev, Eugene
Zuin, Stefano
Zych, Eugeniusz

